# Three-dimensional intracardiac echocardiography and pulmonary embolism

**DOI:** 10.1186/s12947-020-00220-3

**Published:** 2020-08-20

**Authors:** Konstantin Yastrebov, Laurencie Brunel, Hugh S. Paterson, Zoe A. Williams, Paul G. Bannon

**Affiliations:** 1 Sydney Core Research Facility, University of Sydney, Hybrid Theatre, Charles Perkins Building, Johns Hopkins Dr, Camperdown, Sydney, NSW 2006 Australia; 2 University of New South Wales, Kensington, Sydney, Australia; 3 The Baird Institute of Applied Heart and Lung Surgical Research, Sydney, Australia; 4 Institute of Academic Surgery, Royal Prince Alfred Hospital and University of Sydney, Sydney, Australia

**Keywords:** Three dimensional intracardiac echocardiography, Pulmonary embolism

## Abstract

**Background:**

Three-dimensional intracardiac echocardiography (3D ICE) with wide azimuthal elevation is a novel technique performed for assessment of cardiac anatomy and guidance of intracardiac procedures, being able to provide unique views with good spatial and temporal resolution. Complications arising from this invasive procedure and the value of 3D ICE in the detection and diagnosis of acute cardiovascular pathology are not comprehensively described. This case illustrates a previously unreported iatrogenic complication of clot displacement from the intra-vascular sheath upon insertion of a 3D ICE catheter and the value of 3D ICE in immediate diagnosis of clot in transit through the heart with pulmonary embolism.

**Case presentation:**

We conducted a translational study of 3D ICE with wide azimuthal elevation to guide implantation of a left ventricular assist device (Impella CP®) in eight adult sheep. A large-bore 14 Fr central venous sheath was used to enable right atrial and right ventricular access for the intracardiac catheter. Insertion of the 3D ICE catheter was accompanied by a sudden severe cardiorespiratory deterioration in one animal. 3D ICE revealed a large highly mobile mass within the right heart chambers, determined to be a clot-in-transit. The diagnosis of pulmonary clot embolism resulting from the retrograde blood entry into the large-bore sheath introducer, rapid clot formation and consequent displacement into venous circulation by the ICE catheter was made. The sheep survived this life-threatening event following institution of cardiovascular support allowing completion of the primary research protocol.

**Conclusion:**

This report serves as a serious warning to the researchers and clinicians utilizing long large-bore sheath introducers for 3D ICE and illustrates the value of 3D ICE in detecting clot-in-transit within right heart chambers.

## Background

Intracardiac echocardiography (ICE) is increasingly used for diagnostic procedures and imaging guidance during interventions. It offers unique high-resolution views of cardiac anatomy and intracardiac devices. The intracardiac echocardiographic catheter is inserted through a vascular sheath following central venous catheterization [1]. The tip of the ICE catheter is usually positioned within right heart chambers and coronary sinus [2], although left atrial imaging is possible after atrial septal puncture [3].

Contemporary two-dimensional (2D) ICE catheters have a diameter of 8Fr and a length of 90 cm. Three-dimensional (3D) ICE catheters have a diameter up to 12.5 Fr and are 90 cm in length. 3D ICE catheters provide a 90° imaging sector and are commercially available in narrow-angle (22°) [4] and wide-angle (up to 50°) azimuthal elevation [5]. The central venous sheath introducer is usually a single-lumen vascular catheter with the inner bore diameter larger than the ICE catheter, incorporating a hemostatic valve and a side port. The ICE catheter is introduced into circulation via the hemostatic valve and advanced under live ultrasound imaging into the right heart chambers with imaging obtained using manual rotation and mechanical steering of the ICE catheter tip.

There is limited world-wide clinical experience [6] and a paucity of described complications associated with the use of wide-angle azimuthal elevation 3D ICE.

We conducted a translational research project to assess the implantation of the left ventricular assist device Impella CP® guided by 3D ICE using an adult ovine model [7]. The presently described complication associated with wide-bore sheath introducers used for 3D ICE observed in one of the sheep, serves as a warning to the clinicians and researchers and provides lessons to improve safety. For the first time, this report confirms the feasibility of 3D ICE to provide high-quality diagnostic imaging of right heart clot-in-transit.

## Case presentation

The study was approved by the University of Sydney (Australia) Animal Research Ethics Committee (2019/1650 amendment) and conducted at the Charles Perkins Centre for Research, The University of Sydney (Sydney, Australia). It was carried out in accordance with the National Institutes of Health guide for the care and use of Laboratory animals (NIH Publications No. 8023, revised 1978) and in accordance with the relevant guidelines and regulations. Sheep number three of eight developed the reported complication.

### Animal and anaesthesia

This adult female cross merino sheep was acclimatised for at least two weeks prior to the procedure and received routine preventative treatments prior to arrival. The sheep was anaesthetised and mechanically ventilated in synchronized intermittent positive pressure ventilation mode with positive end-expiratory pressure of 5 cm H_2_O monitored by ventilatory pressures, continuous pulse oximetry and end tidal CO_2_. The sheep was in the right lateral position for a left thoracotomy. Invasive monitoring of arterial pressure, central venous pressure and cardiac output were continuously recorded. The anaesthetised sheep was euthanised on completion of the study.

### Echocardiography and central venous cannulation

A physician qualified in transthoracic echocardiography (Advanced Transthoracic Echocardiography training, Level 3) [8] performed all echocardiographic examinations (SC2000, Siemens Healthcare GmbH, Erlangen, Germany).

A 14 Fr, 20 cm valved intra-vascular sheath (Cook Medical, Bloomington In, USA) was used for the intracardiac catheter access. A cut-down technique was used to expose the left internal jugular vein and the sheath was inserted by a modified Seldinger technique with a standard dilator. The dilator was removed immediately after intravenous placement of the vascular sheath. The sheath was sutured to the skin.

For the initial echocardiographic assessment, an AcuNav Volume 12.5 Fr, 90 cm four-ways steerable ICE catheter (Siemens Medical Solutions, USA Inc., Mountain View, CA) was inserted through the haemostatic valve of the vascular sheath approximately 5 min following removal of the dilator during which time systemic heparinisation had not been administered until immediately prior to the ICE catheter insertion.

Live three-dimensional volumes were acquired at 6 Mhz fundamental and 8 Mhz harmonic frequencies with the sector set at 90 × 50° as standard and adjusted together with the depth as required. Maximum achievable frame rate was 14–20 frames per second for 3D grey scale imaging. Images were subsequently cropped and processed using on-cart software.

Sudden profound arterial oxygen desaturation was detected following insertion of the 3D ICE catheter via the sheath into the superior vena cava.

**Fig. 1:**
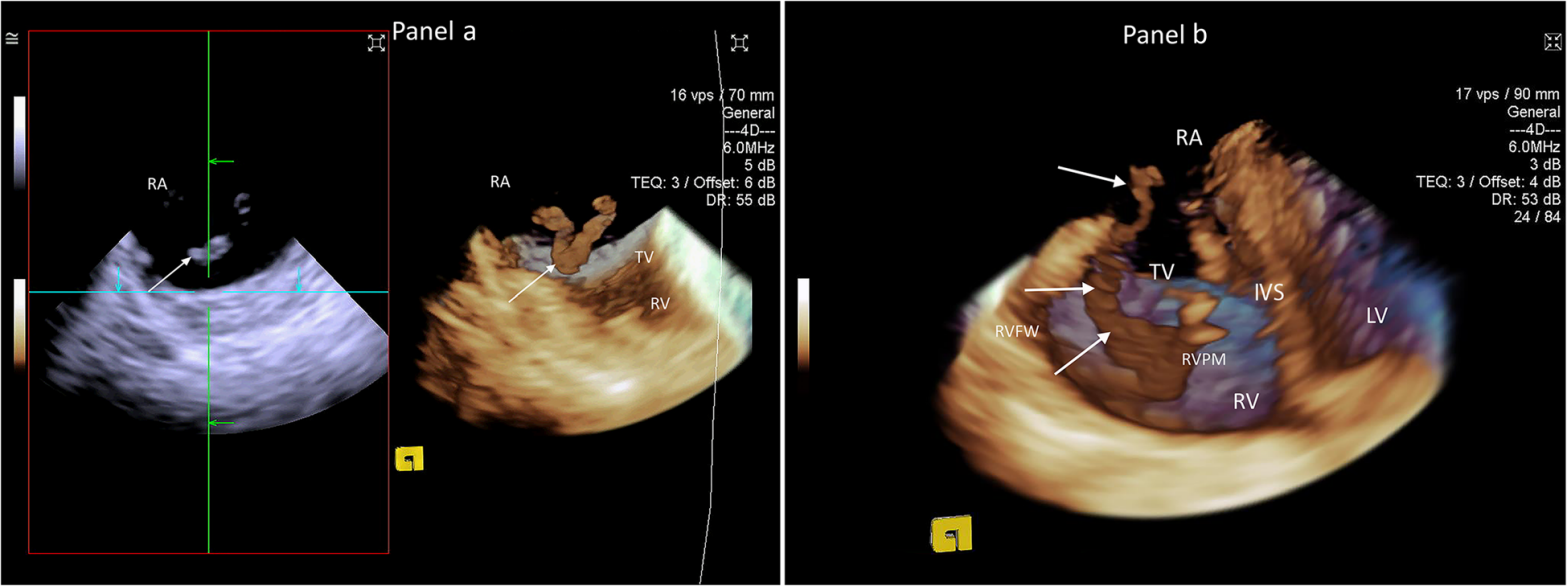
Right atrial and right ventricular clot-in-transit demonstrated with 3D ICE. Panel **a**: Right atrial mobile clot (white arrow) in two-dimensional and three-dimensional intracardiac echocardiography imaging. 3D ICE offers significantly better spatial appreciation of the clot size and position inside cardiac chamber. RA indicates right atrium; TV, tricuspid valve; RV – right ventricle. Panel **b**: Large mobile clot (white arrows) is clearly demonstrated in three-dimensional intracardiac echocardiographic image within right atrial and right ventricular cavities. It is prolapsing through the tricuspid valve towards right ventricular apex. The right ventricle is dilated with the interventricular septum bowing to the left. RA indicates right atrium; RV, right ventricle; TV, tricuspid valve; LV, left ventricle; RVFW, right ventricular free wall; RVPM, right ventricular papillary muscle

A 3D ICE right atrial view in live full volume modality and in the default ICE catheter position initially revealed a large mobile mass (white arrows) within the right atrium (Fig. 1 Panel a). Minor retroflection of the intracardiac catheter provided a clear and complete view (Fig. 1 Panel b, Movie 1 in Additional Files) of the mass traversing the tricuspid valve from the right atrium into a severely dilated right ventricle. The mass stretched along the right ventricular free wall to the base of the prominent right ventricular papillary muscle. The interventricular septum was bowing towards the left ventricle, creating an abnormally globular right ventricular shape. Severe dilatation of the right ventricle and pulmonary artery was noted on direct inspection of the heart via the thoracotomy. The diagnosis of pulmonary embolism with reactive pulmonary arterial vasoconstriction with clot in-transit visualized within the right heart chambers was made. The mass disappeared several minutes later coinciding with further severe haemodynamic compromise, which suggested clot migration into pulmonary circulation, causing progressive pulmonary embolism. Severe arterial hypotension and cardiac output reduction gradually improved with boluses of adrenaline, initiation of noradrenaline and a titrated dobutamine infusion. Full systemic anticoagulation was achieved with administration of heparin (5000 units). Thrombolysis was not considered in a thoracotomised animal.

## Discussion

Intracardiac echocardiography is mostly used by electrophysiologists and interventional cardiologists. Its proximity to the cardiac structures creates unique imaging capabilities for the assessment of cardiac anatomy, intracardiac masses and procedural guidance in interventional cardiology [4]. However, the technique is invasive, which increases the risk of iatrogenic complications.

3D ICE with wide azimuthal elevation offers unique incremental value over 2D ICE by volumetric display and spatial imaging relationship between complete cardiac structures, intracardiac masses and devices. The trade-off is that 3D ICE catheters have larger diameters and require larger central venous sheath introducers. Premature removal of the dilator from the large-bore sheath following its insertion and inadequate flushing of the sheath with crystalloid solution may lead to the retrograde blood entry into the bore. In the absence of adequate systemic anticoagulation, this can predispose to the rapid formation of sizeable clots within the sheath and the potential for clot migration into pulmonary arterial tree. Clot displacement from the sheath will occur upon insertion of the ICE catheter. To our knowledge, such complication has not been previously described. Our report suggests that life-threatening pulmonary embolism should be considered among other diagnoses in patients undergoing ICE who develop sudden episodes of desaturation or significant haemodynamic compromise.

Sheep have a clotting time similar to humans [9, 10], thus making our report highly applicable for human clinical practice. The risk of retrograde blood entry with consequent rapid clot formation inside the large-bore intra-vascular sheaths used for ICE can be potentially reduced by leaving the dilator within the sheath until immediately prior to insertion of the ICE catheter, flushing of the sheath with a heparinised crystalloid solution following removal of the dilator and minimizing the time between vascular sheath placement and insertion of the ICE catheter. Systemic anticoagulation and continuous infusion of heparin solution via the side-port of vascular introducer should be considered when using large-bore vascular sheaths. Deviation from our usual practice of administering systemic heparinization and insertion of ICE catheter immediately following placement of the sheath introducer contributed to the described complication.

An ability of ICE to identify formed thrombi in the left atrial cavity [11], the right ventricular apex [12], and the pulmonary artery [13] has been previously reported during ICE-guided electrophysiology ablation procedures. This is, to our knowledge, the first report of clot-in-transit being imaged with 3D ICE in the right heart chambers. Intimate proximity of ICE transducers to the structures of interest allows high frequency ultrasound scanning owing to the excellent spatial resolution. 2D echocardiographic identification and comprehensive assessment of intracardiac thrombi can be inadequate due to the potential for two-dimensional planes missing or under-estimating complex three-dimensional long mobile structures. 3D wide azimuthal ICE allows real-time display of the complete cardiac structures, even relatively close to the transducer. This case confirms the feasibility of 3D ICE for immediate definitive diagnosis of clot-in-transit within right heart chambers, thus offering early initiation of life-saving therapy. It further demonstrates the exceptional technical capability of the contemporary 3D ICE to provide a uniquely detailed demonstration of the morphological and anatomical relationships between the clot and the cardiac structures, with high temporal resolution.

## Conclusion

This report highlights the newly identified risk of a potentially life-threatening complication during ICE resulting from rapid clot formation within a long large-bore central intra-venous sheath and consequent thrombus displacement into the pulmonary circulation during insertion of the ICE catheter. 3D ICE with wide azimuthal elevation demonstrated excellent diagnostic capabilities to image the clot in-transit within right heart chambers.

## Supplementary information

**Additional file 1.**

## Data Availability

The datasets acquired during this study are available from the corresponding author on reasonable request.

## Abbreviations

2D: two-dimensional
3D: three dimensional
ICE: intracardiac echocardiography
